# An Unusual Case of Stridor: Severe Tracheal Narrowing Secondary to Esophageal Food Impaction

**DOI:** 10.5811/cpcem.2019.5.43027

**Published:** 2019-07-22

**Authors:** Ryan McCreery, Matthew Meigh

**Affiliations:** * Orange Regional Medical Center, Department of Emergency Medicine, Middletown, New York; † New York Colleges Osteopathic Medicine Educational Consortium, Department of Emergency Medicine, Middletown, New York

## Abstract

Foreign body impaction (FBI) in the esophagus has the potential to be a serious condition with a high mortality rate. Although the majority of foreign bodies trapped within the esophagus pass spontaneously, some do require endoscopic intervention. This case discusses a 95-year-old-female with a history of cerebral vascular accident who presented with acute onset respiratory distress with inspiratory stridor. The patient denied any episodes of choking or foreign body sensation. Further imaging revealed a large food bolus within the esophagus with extensive tracheal narrowing. The patient was diagnosed promptly and successfully managed endoscopically. This case presentation emphasizes the need to maintain a high index of clinical suspicion for FBI in high-risk populations, especially when the patient’s history makes it unlikely. In the setting of respiratory complications, airway protection remains a priority, but an accurate diagnosis with timely intervention is paramount.

## CASE PRESENTATION

A 95-year-old female with history of cerebral vascular accident presented to our emergency department for acute onset respiratory distress with inspiratory stridor. Vital signs revealed the patient to be hypertensive, tachycardic and tachypneic with intermittent episodes of mild hypoxia. On physical exam, she was unable to phonate with audible stridor but without drooling or trismus. Lung sounds were clear to auscultation bilaterally. She denied any choking or foreign body sensation. Radiography of the soft tissue of the neck and chest were unremarkable. Bedside transnasal flexible laryngobronchoscopy was normal to the level of the cords.

**Image 1:**
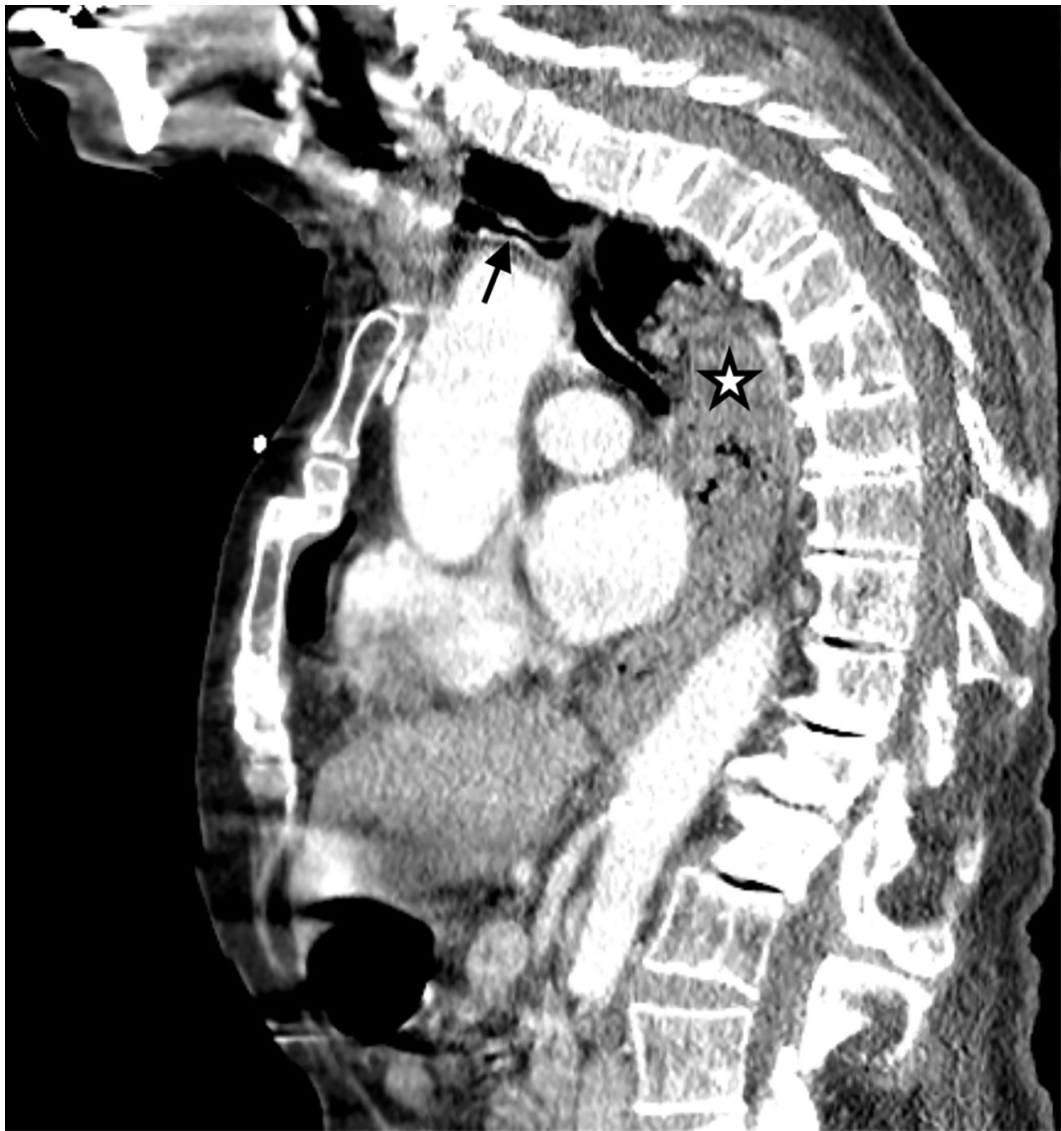
Computed tomography (sagittal view) demonstrating luminal narrowing of the trachea (black arrow) with marked distention of the esophagus. Significant inspissated material noted throughout the distended esophagus (white star).

**Image 2:**
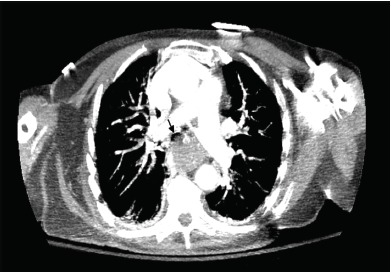
Computed tomography (axial view) demonstrating near obliteration of the trachea at the level of the manubrium secondary to esophageal distention (black arrow).

Contrast-enhanced computed tomography (CT) chest revealed significant tracheal compromise from marked distention of the esophagus (Images 1 and 2). The patient was intubated and underwent an emergent therapeutic bronchoscopy and esophagogastroduodenoscopy (EGD). While bronchoscopy was unremarkable, EGD showed a large food bolus extending from the upper to lower esophageal sphincters. Food bolus removal resolved her respiratory symptoms, and subsequent esophagram showed marked esophageal dysmotility.

## DISCUSSION

Esophageal foreign body impaction (FBI) can be a serious condition carrying high mortality when not properly diagnosed or if management is delayed. While 80–90% of FBIs spontaneously pass from the esophagus to the stomach, an estimated 10–20% require endoscopic intervention.^1^ Life-threatening complications include airway compromise and esophageal wall perforation, which can lead to mediastinitis, fistula, abscess, empyema, or death.^2^ In adults, stridor due to esophageal FBI is a rare complication that not only requires airway protection but an accurate diagnosis with timely intervention.^3^

**CPC-EM Capsule**

What do we already know about this clinical entity?

In the setting of respiratory compromise, an accurate diagnosis of esophageal foreign body impaction with timely intervention is paramount to reduce mortality.

What is the major impact of the image(s)?

These images clearly depict severe tracheal narrowing from esophageal food impaction and help raise awareness for this particular process.

How might this improve emergency medicine practice?

Consider esophageal food impaction in the differential diagnosis for stridor in the awake, adult patient without localizing signs or symptoms of dysphagia.
